# Stigmatizing Language for Alcohol Use Disorder and Liver Disease on Liver Transplant Center Websites

**DOI:** 10.1001/jamanetworkopen.2023.55320

**Published:** 2024-02-08

**Authors:** Rachael Mahle, Adedayo Okanlawon, Jay Luther, Jeremy Louissaint, Wei Zhang

**Affiliations:** 1 Department of Medicine, Massachusetts General Hospital, Boston; 2 Gastroenterology Unit, Massachusetts General Hospital, Boston; 3 Alcohol Liver Center, Massachusetts General Hospital, Boston; 4 Harvard Medical School, Boston, Massachusetts; 5 Division of Digestive and Liver Diseases, University of Texas Southwestern Medical Center, Dallas

## Abstract

This cross-sectional study investigates the adoption of recommendations for the use of nonstigmatizing language to describe alcohol use disorder and alcohol-related liver disease among liver transplant centers in the US.

## Introduction

Stigma associated with alcohol use disorder (AUD) and alcohol-associated liver disease (ALD) can cause delayed disease detection and intervention and may influence liver transplant allocation.^1^ Although multiple societies recommended use of nonstigmatizing language,^2,3,4^ it is unclear how well these recommendations are followed in materials from transplant centers. Although stigma surrounding AUD and ALD is multifaceted, we investigated the adoption of these language recommendations among liver transplant centers in the US.

## Methods

We conducted a cross-sectional study systematically reviewing accredited US liver transplant centers and addiction psychiatry websites at the same institutions. Pediatric centers were excluded due to their low ALD rates. These websites were evaluated by 2 independent reviewers (R.M. and A.O.) for use of stigmatizing language (“alcoholism,” “alcoholic,” “alcohol abuse”) and nonstigmatizing language (“alcohol use disorder,” “alcohol misuse,” “alcohol associated,” “alcohol related”). Websites using both types were categorized as using mixed language. Language use was analyzed with a 2-sided χ^2^ test with *P* < .05 considered significant, and interrater reliability was measured with the Cohen κ. The STROBE reporting guideline was followed. Data were obtained from public websites, and no institutional review board review was needed per the Common Rule.

## Results

Of 114 liver transplant centers reviewed, 82 (71.9%) described 1 or more of the following: AUD, ALD, alcohol-associated hepatitis, and alcohol-associated cirrhosis. Of the 104 addiction psychiatry websites reviewed, 39 (37.5%) described AUD while none mentioned liver diseases. The Cohen κ coefficient was 0.731. Seventy-two of the 82 transplant websites (87.8%) and 18 of the 39 addiction psychiatry websites (46.2%) used stigmatizing language. For AUD, only stigmatizing language was used 79.2% of the time (42 of 53) compared with 20.8% (11 of 53) for only nonstigmatizing language and 0% for mixed language. This was significantly higher than on addiction psychiatry websites, which used only stigmatizing language at a rate of 30.8% (12 of 39; *P* < .001). Of 60 centers that described AUD on their own and corresponding addiction psychiatry department websites, 19 (31.7%) had consistent language use between the 2. For ALD (n = 60), 66.7% of websites (n = 40) used only stigmatizing language, 20.0% (n = 12) used only nonstigmatizing language, and 13.3% (n = 8) used mixed language. When discussing alcohol-associated hepatitis (n = 47), 95.7% of websites (n = 45) used only stigmatizing language, while 2.1% (n = 1) used only nonstigmatizing language, and 2.1% (n = 1) used mixed language. For alcohol-associated cirrhosis (n = 28), 85.7% of websites (n = 24) used “alcoholic cirrhosis,” while 10.7% (n = 3) consistently used nonstigmatizing language and 3.6% (n = 1) used mixed language (Figure).

**Figure.**
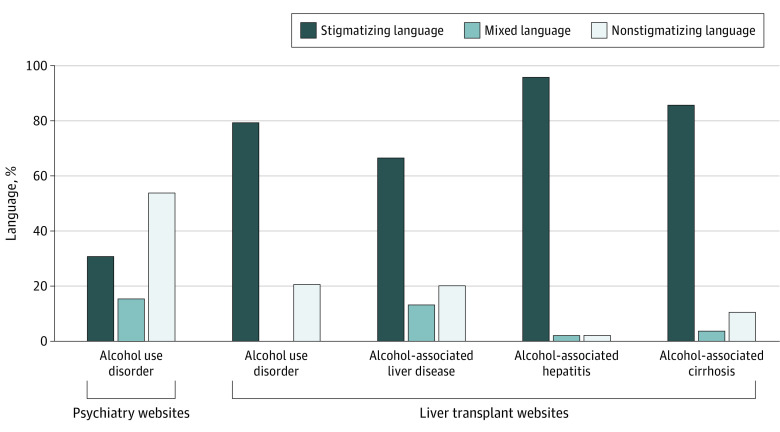
Use of Stigmatizing Language on Liver Transplant Center and Addiction Psychiatry Websites

## Discussion

Our findings reveal limited and inconsistent adoption of nonstigmatizing language for AUD and ALD across transplant websites compared with addiction psychiatry websites within the same institutions, suggesting the variability is not due to a system-wide policy but is center specific. This disparity underscores the need for the use of more patient-centered language in transplant centers to reduce stigma. The slow adoption of nonstigmatizing language may be due to a lack of awareness about its association with health care and resistance to change. Patients increasingly seek health-related information online, which can significantly influence behavior.^5,6^ Although it is encouraging that academic societies advocate for nonstigmatizing language to describe ALD, our results call for hospital systems to reassess and update their language to align with nonstigmatizing terminology, which may enhance patient care and improve patients’ willingness for treatments.

This study’s limitations include the challenge in defining stigmatizing language comprehensively. Although we did not encompass all stigmatizing language for ALD, our study shows more frequent use of terms such as *alcoholic*, *alcoholism*, and *alcohol abuse* on transplant websites compared with psychiatry counterparts. These findings highlight the need for further research on the association of language with ALD perception and treatment.

## Notes

Supplement.
Data Sharing Statement
