# Supplementary material for: Stigmatizing Language for Alcohol Use Disorder and Liver Disease on Liver Transplant Center Websites
Source: JAMA Netw Open. 2024 Feb 8;7(2):e2355320. doi: 10.1001/jamanetworkopen.2023.55320 (PMC10853823; doi:10.1001/jamanetworkopen.2023.55320)
Supplement: Supplement. — Data Sharing Statement [file jamanetwopen-e2355320-s001.pdf]

# Data Sharing Statement

Mahle. Stigmatizing Language for Alcohol Use Disorder and Liver Disease on Liver Transplant Center Websites. *JAMA Netw Open*. Published February 08, 2024.
doi:10.1001/jamanetworkopen.2023.55320

**Data**
**Data available:** Yes
**Data types:** Data (not involving human participants)
**How to access data:** wzhang50@mgh.harvard.edu
**When available:** With publication

**Supporting Documents**
**Document types:** None

**Additional Information**
**Who can access the data:** researchers whose proposed use of the data has been approved
**Types of analyses:** for any purpose
**Mechanisms of data availability:** after approval of a proposal
